# A roadmap to establish a comprehensive platform for sustainable manufacturing of natural products in yeast

**DOI:** 10.1038/s41467-023-37627-1

**Published:** 2023-04-06

**Authors:** Gita Naseri

**Affiliations:** 1 Max Planck Unit for the Science of Pathogens, Charitéplatz 1, 10117 Berlin, Germany; 2 Institut für Biologie, Humboldt-Universität zu Berlin, Philippstrasse 13, 10115 Berlin, Germany

**Keywords:** Metabolic engineering, Synthetic biology, Applied microbiology, Natural product synthesis

## Abstract

Secondary natural products (NPs) are a rich source for drug discovery. However, the low abundance of NPs makes their extraction from nature inefficient, while chemical synthesis is challenging and unsustainable. *Saccharomyces cerevisiae* and *Pichia pastoris* are excellent manufacturing systems for the production of NPs. This Perspective discusses a comprehensive platform for sustainable production of NPs in the two yeasts through system-associated optimization at four levels: genetics, temporal controllers, productivity screening, and scalability. Additionally, it is pointed out critical metabolic building blocks in NP bioengineering can be identified through connecting multilevel data of the optimized system using deep learning.

## Abstract (web summary)

Yeasts, *Saccharomyces cerevisiae* and *Pichia pastoris*, are promising chassis for the production of nature products (NPs). Here, the author discusses establishing a comprehensive platform for sustainable production of NPs via system-associated optimization at genetics, temporal controllers, productivity screening, and scalability levels.

## Introduction

More than 50% of small-molecule therapeutic agents in current clinical usage are natural products (NPs)^1^. Importantly, NPs have been sources of new drugs over the past four decades^2^, and are used as prototypes to discover new drugs because of their massive scaffold diversity and structural complexity^3^. Plants are excellent producers of NPs with diverse biological activities^4,5^. Currently, the principal source of most NP-derived pharmaceuticals remains direct extraction from plants^3^. However, the lack of continuous availability of sufficient amounts of natural sources and variations in yields have limited NP extraction^5^. Considering the pressures of food security and population ageing^6^, alternative approaches are urgently needed to enable environmentally friendly production of NP drugs.

Thanks to the rapid progress of synthetic biology, the microbial synthesis of more complex NPs has been possible. A prime example is the successful construction of a yeast microbial cell factory for strictosidine and its downstream vinblastine, positioning yeast as a scalable system to produce more than 3000 reported NPs^7^. However, only a few natural compounds produced by microbes have reached commercial scale^8,9^. Opportunely, the increasing sizes of multi-omics datasets and the acceleration of technologies supporting structural analysis and enzyme characterization, the time to discovery of enzymatic steps for synthesis of complex NPs in plants has decreased^10^. To capitalize on this opportunity, it’s urgent to establish a standardized chemical engineering approach. Here, I discuss yeast as a microbial cell factory for production of complex plant NPs with pharmaceutical importance, such as alkaloids^11^ and flavonoids^12^.

## Yeast for the heterologous production of NPs

Multi-purpose yeasts, including *Saccharomyces cerevisiae* and *Pichia pastoris* (syn. *Komagataella phaffii*), are the best characterized and the most widely used microorganisms in the fermentation industry^13^. They can potentially resolve issues associated with low heterologous NP product yield observed in other expression hosts, such as *E. coli* and mammalian systems^14^. Yeast is able to synthesize the required precursors of diverse plant NPs including alkaloids, an imperative group of NPs containing at least one nitrogen atom^15^. Yeast expresses endoplasmic reticulum (ER)–localized cytochrome P450 monooxygenases (P450s, also called CYPs), membrane-anchored cytochrome P450 reductases (CPRs), scaffold Pictet-Spenglerases, and berberine-bridge enzymes, which are essential enzymes for producing NPs^16,17^. Yeast, like higher eukaryotic organisms, is equipped with subcellular organelles, such as mitochondria, that have been employed to shield the heterologous enzymes^18^. Compared to mitochondria, peroxisomes are more appropriate organelles for the compartmentalization of engineered NP pathways. Peroxisomes are detoxifying organelles with greater ability than other organelles to tolerate and sequester toxic molecules such as NPs^19^. Moreover, they are near the ER, thus facilitating efficient cytochrome P450-mediated oxidation of the intermediates required to produce NPs such as monoterpene alkaloids^20^.

The *S. cerevisiae* is typically used as a host for manufacturing complex NPs^7,21^. Recently, the non-conventional methylotrophic yeast *P. pastoris* has emerged as an attractive host for NP production^22^. *P. pastoris* has the advantages of methanol utilization, compatibility with higher density cell growth and large-scale fermentation. Furthermore, it shows a higher expression level of heterologous protein than *S. cerevisiae*. For example, recombinant proteins expressed under the control of the yeast alcohol oxidase 1 (*AOX1*) promoter can contribute more than 30% of the total proteins in *P. pastoris*^23^. A recent study has shown that a cytochrome P450 enzyme, which catalyzes the conversion of scoulerine (a product downstream of reticuline) to cheilanthifoline, exhibits a higher conversion rate in *P. pastoris* cells (70%) than *S. cerevisiae* cells (20%)^24^. Interestingly, when *P. pastoris* is grown on methanol, its peroxisomes substantially multiply^25^, which can be deployed to increase the production of heterologous enzymes.

**Fig. 1:**
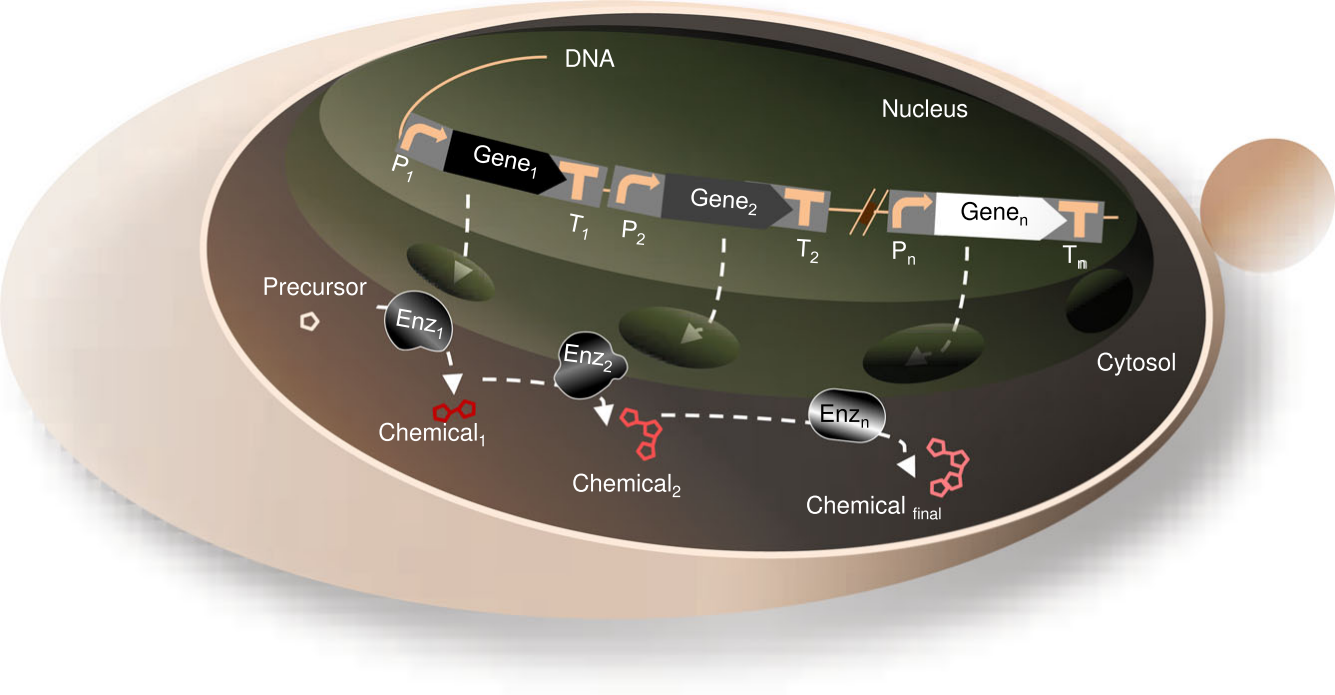
Schematic overview of the principle of heterologous chemical production in yeast. The DNA sequences of genes and regulatory elements, promoters, and terminators (to, respectively, start and terminate the transcription of the individual genes) are engineered into the DNA located inside the nucleus in the cell. Followed by translation using the host translational machinery, the expressed enzymes convert the yeast precursor to the intermediate chemicals and, subsequently, the chemical of interest. The schematic shows a chemical biosynthetic pathway consisting of genes ‘Gene_1_’, ‘Gene_2_’, and ‘Gene_n_’, requiring three promoters ‘*P*_*1*_’, ‘*P*_*2*_’, and ‘*P*_*n*_’, and three terminators ‘*T*_*1*_*’*, ‘*T*_*2”*_, ‘*T*_*n*_*’*). Enz enzyme, *P* promoter, *T* terminator.

The genes coding for NPs are typically randomly distributed through the genome in the native host^26^. To enable yeast cells to produce a chemical-of-interest (Fig. 1), each individual gene encoding one enzyme of the biosynthetic pathway or biosynthesis gene clusters needs to be expressed from the individual promoter and terminator^27^. Recently several advanced tools such as inducible synthetic transcription factors (synTFs) and promoters^28,29^, terminators^30^, and genome editing tools^31^ have been developed to facilitate regulation of heterologous protein expression in *S. cerevisiae*. Fortunately, these tools have been found to be functional in tuning heterologous protein expression levels in *P. pastoris*, for which a limited number of tools and methods are available for metabolic engineering. Considering the dual application of engineering tools and methods in both chassis’ organisms, *P. pastoris* has attracted attention as an ideal platform for metabolic engineering of plant NPs.

## Steps to establish yeast as a sustainable platform for NP production

**Fig. 2:**
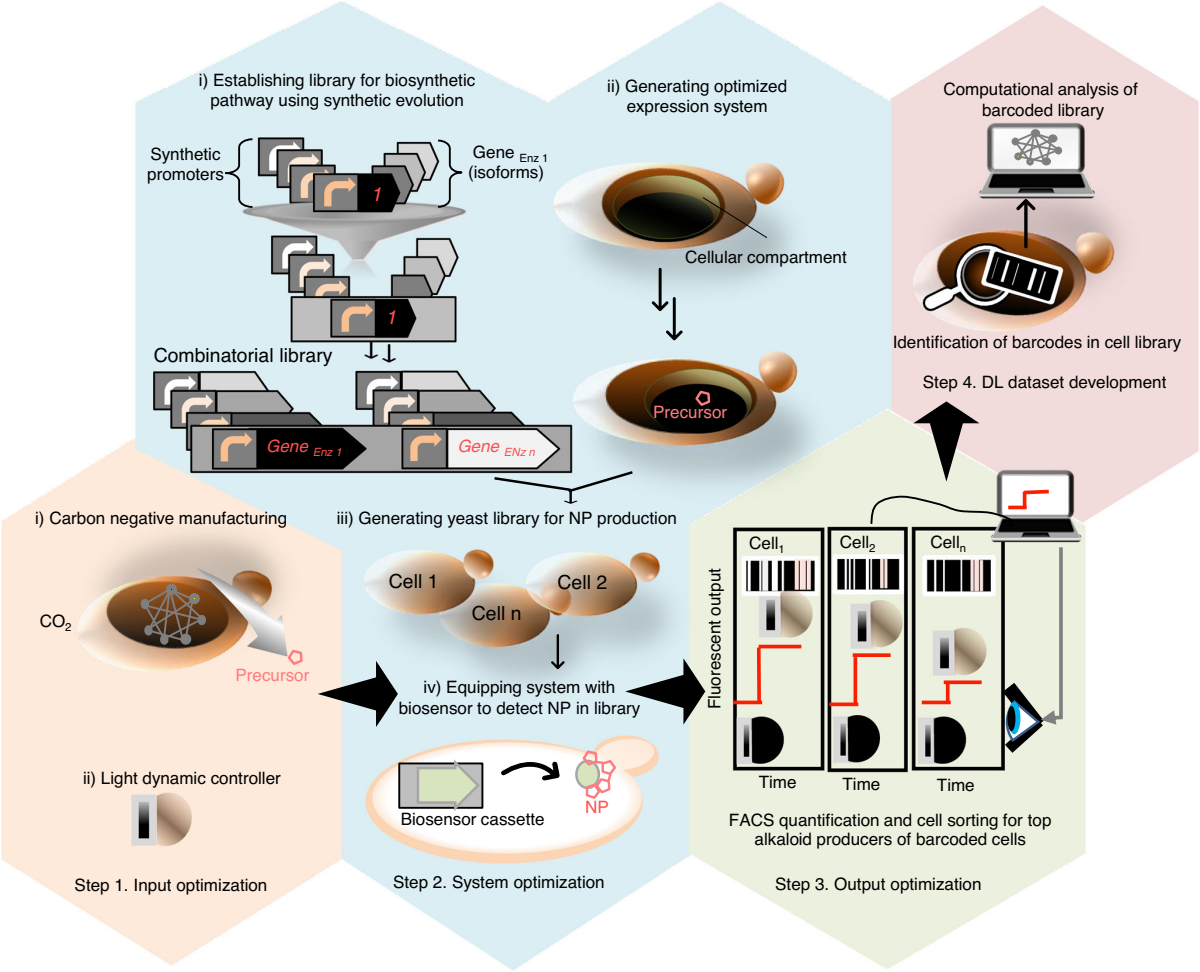
Schematic overview of the platform for sustainable production of NPs in yeast. To sustainably produce the highly required pharmaceutical NPs in an environmentally friendly manner, a four-step platform is designed. Step 1, input optimization (orange box): For carbon-negative manufacturing (i), a photosynthetic microorganism or engineered yeast fixes CO_2_.The fixed carbon can provide yeast with the required sources for production NP precursors. A light-inducible system is used to dynamically control NP metabolite production in yeast (ii). Step 2, system optimization (blue box): The library of light-inducible synthetic regulators is assembled upstream of genes of enzyme isoforms by using combinatorial assembly approaches (i). Protein engineering strategies are applied to design enzyme derivatives with enhanced activity. Next, yeast cells with rewired endogenous metabolites for the enhanced production of the required precursor in peroxisomes (blue box, ii) are chromosomally engineered with the pathway library (iii). The presence of the NP-responsive biosensor in the yeast cell enables rapid detection of NP production (iv). Step 3, output optimization (green box): Cells with a high level of fluorescence output are subjected to automated process optimization, and the top producers are sorted with FACS flow cytometry. Step 4, DL dataset development (red box): The library variants are subjected to single-cell droplet-based transcriptomics and NGS analyses to feed the DL dataset. The multilayer metadata obtained from dynamic optogenetic stimulation, the productivity of library members and growth fitness in real-time, and the genetic identity of low-to-top producers are used to fit model parameters and extract models to predict optimal conditions for heterologous production of other NPs. To simplify the figure, only three cells, Cell 1, Cell2, and Cell n, from the combinatorial library were shown. CO_2_ carbon dioxide, DL deep learning, FACS fluorescence-activated cell sorting, NGS next-generation sequencing, NP natural product.

To increase the opportunities for carbon-neutral or carbon-negative manufacturing, the core metabolism of yeast needs to shift from its dependence on sugars to one-carbon compounds. To this end, the yeast NP production platform can be potentially equipped with two optimizable inputs: (i) one-carbon (C_1_) resources, e.g., carbon dioxide (CO_2_); and (ii) an optogenetic inducer (Fig. 2, Step1). The fixed C_1_ can provide yeast the required carbon sources for NP production. Then, light can activate the transcription of synthetic regulators that control the expression of the NP biosynthetic pathway genes in engineered yeast (Fig. 2, Step 2). To adjust the regulation of intermediate metabolites, many diverse synthetic transcriptional units for each individual gene, consisting of synTFs and pathway genes, should be created through combinatorial optimization methods. Next, the library of synthetic transcriptional units is engineered into the genome of host cells. Compartmentalization of the precursor biosynthetic pathways in yeast organelles allows for increased production of the required substrate and its efficient channeling to downstream enzymes for NP biosynthesis^32^. Screening the generated cell library with biosensors allows for high-throughput quantification of NP production in yeast cells. The fermentation conditions and light induction system must be optimized in the automated bioreactors to increase the output of the entire system (Fig. 2, Step 3). The high-throughput computational sequencing analysis of the top members of the yeast combinatorial library that are labeled with barcodes is used to feed the deep learning (DL). The obtained dataset drives biosynthetic pathways navigation for increased production of diverse natural small molecules in heterologous hosts (Fig. 2, Step 4). The following sections review current and emerging technologies to conceptually advance the individual steps of the roadmap and align them to illustrate the platform described in Fig. 2.

## Input optimization

Yeast tends to offer modest emissions savings over conventional agricultural processes in the production of pharmaceutical chemicals, including less consumption of water, nutrients, energy, and land, lower costs, faster and more stable production cycles, and higher quality products^33^. However, the cost and land resources required to produce sugar feedstocks make manufacturing pharmaceutical products in yeast not entirely environmentally friendly. In addition, with rising concerns regarding global climate change, a transition to clean energy sources (e.g., light and hydrogen) to achieve net zero emissions of natural gases will be necessary^34^. In the next three subsections, I discuss strategies for carbon-negative manufacturing through enabling yeast to metabolize CO_2_ or pairing yeast with autotrophic microorganisms, as well as approaches to using light to activate NP production.

### Carbon-negative manufacturing in yeast

To switch to carbon-negative manufacturing, a recent study has suggested a promising metabolic engineering approach by harnessing autotrophic acetogens that uses the Wood-Ljungdahl pathway^35^. However, applying acetogenic bacteria or other natural C_1_-utilizers for industrial-scale production is hindered by the limited availability of genetic manipulation tools, slow growth, and/or a narrow product spectrum^36^. Hence, chemical engineering strategies enabling *S. cerevisiae* and *P. pastoris* to utilize CO_2_, as a carbon source, rather than sugar, are increasingly being considered.

**Fig. 3:**
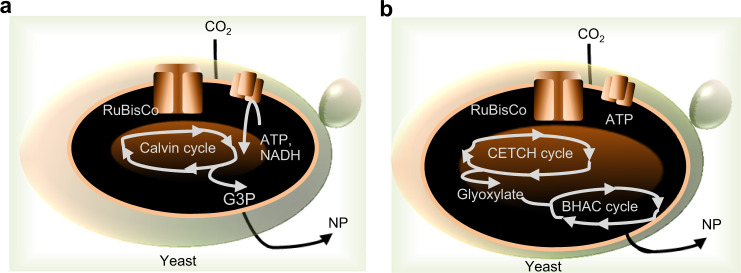
Synthetic routes for carbon-negative manufacturing to produce NP in yeast. **a** The yeast is engineered for the Calvin cycle to convert CO_2_ to G3P, RuBisCo expression, and enhanced production of ATP and NADPH. **b** The yeast is engineered for the synthetic CETCH cycle to produce glyoxylate from CO_2_, the β-hydroxyaspartate cycle to convert glyoxylate to NP, RuBisCo expression, and enhanced production of ATP and NADPH. ATP, adenosine triphosphate, BHAC β-hydroxyaspartate cycle, CETCH crotonyl-coenzyme A (CoA)/ethylmalonyl-CoA/hydroxybutyryl-CoA, CO_2_ carbon dioxide, G3P glyceraldehyde-3-phosphate, NADPH nicotinamide adenine dinucleotide phosphate, NP natural product.

Methylotrophic *P. pastoris* can obtain all the carbon and energy needed for growth and metabolism from reduced C_1_-molecule methanol^37^. However, it leads to release of CO_2_ that cannot be used by *P. pastoris*^37^. By adding eight heterologous genes and deleting three native genes, Gassler et al. have engineered the peroxisomal methanol-assimilation pathway of *P. pastoris* into a CO_2_-fixation pathway resembling the Calvin–Benson–Bassham cycle (hereafter denoted the Calvin cycle)^38^. The Calvin cycle is then targeted to either the cytosol or peroxisome. Next, the team have replaced the formaldehyde-assimilating dihydroxyacetone synthase enzyme of *P. pastoris* with the bacterial ribulose 1,5-bisphosphate carboxylase/oxygenase (RuBisCO) enzyme. After introducing phosphoribulose kinase from spinach, the generated yeast strain directly assimilated CO_2_ instead of methanol, and converted it to organic acids^39^. Portions of the Calvin cycle have been introduced into the yeast *S. cerevisiae*^40^. To enhance CO_2_-fixation efficiency via the Calvin cycle in yeast, several optimization routes, for example, expressing RuBisCo derived from various origins have been evaluated^41^. Li et al. have increased the production of adenosine triphosphate (ATP), and relieved the nicotinamide adenine dinucleotide phosphate (NADPH) imbalance involved in the CO_2_-fixation Calvin cycle^42^. A highly active RuBisCo from the endosymbiont of the deep-sea tubeworm *Riftia pachyptila*^43^ may offer a unique application avenue for creating an efficient artificial photosynthetic pathway. Ultimately, yeast requires conversion of the Calvin cycle’s final product, glycerate 3-phosphate (G3P), to precursors for NP manufacturing (Fig. 3a). Kildegaard et al. have optimized the conversion of G3P to acetyl-CoA, required precursor for production monoterpene indole alkaloids (MIAs), by overexpressing native enzymes and heterologous acetyl-CoA synthase^44^. Combining the above engineering efforts may enable establishment of a yeast strain that can efficiently assimilate CO_2_ into acetyl-CoA-derived NPs via the Calvin cycle. As an alternative to natural CO_2_ fixation, crotonyl-coenzyme A (CoA)/ethylmalonyl-CoA/hydroxybutyryl-CoA (CETCH) has been established for the in vitro fixation of CO_2_^45^. The CETCH cycle, based on crotonyl-CoA carboxylases, allows converting CO_2_ into glyoxylate at a higher carbon fixation rate than the Calvin cycle. CETCH has been successfully coupled to (i) encapsulated photosynthetic membranes for light-powered CO_2_ fixation to glyoxylate^46^, and (ii) the β-hydroxyaspartate cycle, a glyoxylate assimilation cycle from marine bacteria, to convert CO_2_ to acetyl-CoA in vitro^47^. Although light-responsive CETCH cycle has not yet been implemented in vivo, it may offer a way to couple CO_2_ assimilation pathways with NP-producing pathways in yeast (Fig. 3b). CETCH cycle requires two ATPs and three NADPH cofactors to convert two CO_2_ molecules into organic compounds^45^.

CETCH and other routes relying on C1-assimilation require an energy source to convert thermodynamically stable CO_2_. The relatively low-price methanol has been considered a promising energy source for CO_2_ assimilation. To produce the required energy for CO_2_ fixation, methanol is initially oxidized in the peroxisome of *P. pastoris* to hydrogen peroxide (H_2_O_2_), which may lead to oxidative damage to cells, and the toxic formaldehyde^48^. To minimize oxidative damage, balanced expression of the peroxisomal H_2_O_2_-generating oxidases and catalases through combinatorial optimization is an interesting subject for investigation. To decrease the production level of formaldehyde, Gassler et al. have deleted the *AOX1* in *P. pastoris* (formaldehyde can still be synthesized by AOX2)^38^. In fact, a small fraction of the formaldehyde is required to generate energy (and glyceraldehyde-3-phosphate carbon sugar)^38^. Therefore, the balanced expression of formaldehyde synthesizing and consuming enzymes may enhance energy production in *P. pastoris* using methanol. To improve the performance of *P. pastoris* rewired for CO_2_ assimilation and methanol-based energy system, Gassler et al. have enhanced growth of the autotrophic *P. pastoris* by reverse engineering and adaptive laboratory evolution^38,49^. However, to deploy C_1_’s potential for producing complex NPs in yeast, it should be stressed that a long way has to be taken concerning an efficient energy-producing route establishment in yeast. One promising path to circumvent the problem is the electromicrobial conversion of C_1_ sources into NPs using “green hydrogen” energy carriers^50^. Theoretically, in a custom-designed bioreactor, yeast cells engineered to express hydrogen-oxidizing enzymes can consume hydrogen produced by solar electricity to drive CO_2_ fixation. Cheng et al. have established a bacterial mixotrophical CO_2_ assimilation system utilizing hydrogen, generated by hydrolysis of sodium borohydride, as an energy source^51^. This is an important step toward utilizing green hydrogen to support carbon-negative manufacturing in yeast. However, low solubility, poor mass transfer rate, and process safety issues of hydrogen in microbial cultures are the conceivable obstacles. Hopefully, these can possibly be addressed via a systematic design of chemical-biological interface^52^.

### Artificial communities

In engineering the C_1_-fixation pathway to produce NPs in yeast, we must consider that yeast needs to be engineered to express many heterologous enzymes (in addition to C_1_-fixation enzymes), that may cause a metabolic burden on the yeast cell. Division of labor between cells can alleviate the metabolic burden. However, a few artificial cell-cell communication systems in yeast have been developed^53^. Alternatively, the use of autotrophic microorganisms and yeast in synthetic microbial consortia (SMC) divides labor between cells. Recent progress in SMC designing strategies allows the production of value-added chemicals in an environmentally friendly manner. In an established SMC through co-culture of bacteria *Clostridium acetobutylicum* and *C. ljungdahlii*, CO_2_ has been fixed and converted to C_2_-C_4_ alcohols^54^. In another example, a consortium for a cooperative relationship between alginate-utilizing *Vibrio* sp. dhg and 3-hydroxypropionic acid (3-HP)-producing *Escherichia coli* strains has been established, allowing the direct conversion of alginate to 3-HP^55^. For carbon-negative processing, pairwise SMC can be defined between C_1_-consuming autotrophic bacteria and yeast^56^. However, establishing such artificial communities fails in most cases, as one organism dominates in nutrient competition in co-cultivation condition^57^. Molitor et al. have established a two-stage bioprocessing system between acetogenic bacteria *C. ljungdahlii* and *S. cerevisiae*^58^. First, in a bioreactor with a pure culture of *C. ljungdahlii*, CO_2_ with oxygen and nitrogen supplies (sourced by wastewater) has been converted to acetate. Next, in the other bioreactor with a pure culture of yeast, the acetate has been converted to animal feeding protein^58^. In another two-stage system, CO_2_ (mixed with reducing agents carbon monoxide or hydrogen) has been converted into acetic acid using acetogen *Moorella thermoacetica* in the first-stage anaerobic bioreactor^59^. Subsequently, acetic acid has been utilized for lipid production by engineered oleaginous yeast *Yarrowia lipolytica* in the second-stage aerobic bioreactor^59^. Although establishing a two-stage bioprocessing system can overcome the drawbacks of co-culture schemes, the start-up of two connected bioreactors still generates high capital costs.

**Fig. 4:**
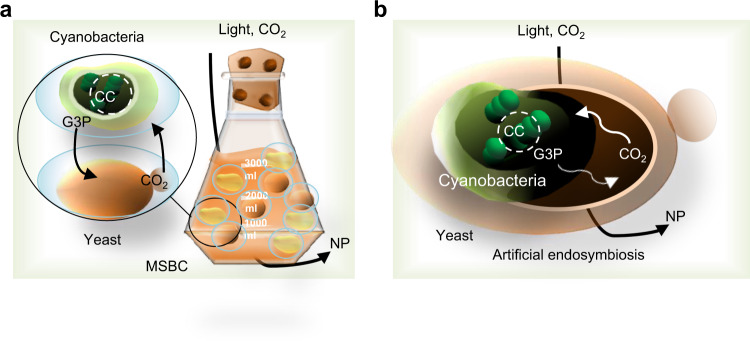
Artificial microbial communities to promote circular acetyl-CoA production in yeast. **a** An MSBC is created in polymeric microcapsules by encapsulation of subpopulations of cyanobacteria and yeast. Phototrophic cyanobacteria capture the CO_2_ in the presence of light. The produced G3P is converted to the required carbon for production of metabolites, including NPs, in yeast. CO_2_ produced by the yeast is reused by the autotrophic partner. **b** Artificial endosymbiosis. Phototrophic cyanobacteria endosymbionts within yeast cells, in which the cyanobacteria perform bioenergetic functions to provide an energy source for the yeast cells to grow and produce chemicals. CC Calvin cycle, CO_2_ carbon dioxide, G3P glyceraldehyde-3-phosphate, MSBC microbial swarmbot consortia, NP natural product.

Alternatively, living materials combining a material scaffold with a porous structure may address the imbalanced co-culture composition issue by enabling the spatial organization of SMC species in co-cultures as that in nature (where spatial organization enables multiple species to coexist)^60^. Recently a methacrylate HEMA-EDMA porous microplate living material has been designed to maximize neighboring well interactions^61^. This culturing system enables exchange of small molecules, but not cells, between wells^61^. With this porous microplate, a spatially segregated co-culture of autotrophic bacteria and yeast can potentially be established. However, extensive follow-up work must be performed on the co-culture of autotrophic bacteria and yeast^61^. Another technique called microbial swarmbot consortia (MSBC) has been created by encapsulating subpopulations in polymeric microcapsules^62^. The cross-linked structure of microcapsules spatially segregates the microbial cells but permits the small-size molecules and proteins to pass. In an MSBC for sustainable production of small NP drugs, photosynthetic *Synechococcus* *elongatus* can capture and convert inorganic CO_2_ to the carbon-based organic compound G3P in the presence of blue light (Fig. 4a). In fact, blue light activates expression of chlorophyll and the key Calvin cycle enzymes. The produced G3P is then taken up by yeast to provide energy and carbon sources^44^. To overcome the futile CO_2_ cycling, which is due to reassimilation of CO_2_ by RuBisCo^63^, the synthetic pathway of malonyl-CoA–oxaloacetate–glyoxylate (employing the efficient phosphoenolpyruvate carboxylase to capture CO_2_)^64^ can be engineered into *S. elongatus* (the autotrophic partner of SMC). Alternatively, through artificial endosymbiosis (Fig. 4b), various mutants of model photosynthetic *S. elongatus* as endosymbionts have been engineered within yeast cells^65^. In principle, the engineered cyanobacteria perform bioenergetic functions to support the growth of yeast cells under optimal photosynthetic growth conditions to produce NPs.

### Solar-powered controller

**Fig. 5:**
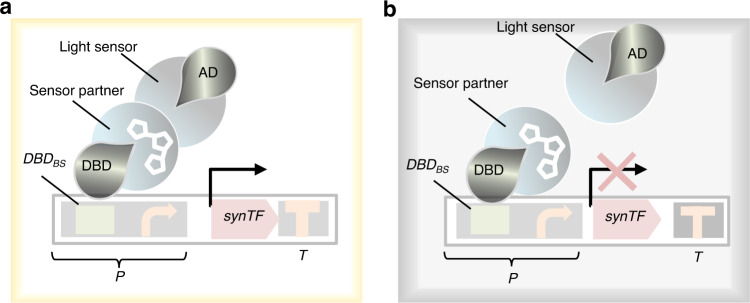
Light-dependent expression system. **a** Light triggers the dimerization of the light-sensing domain and its partner, thus leading to the joining of DBD and AD to form a functional synthetic activator. Next, the synthetic activator targets its *BS* within a promoter, thus resulting in the expression of a synTF. **b** In the absence of light, the synthetic activator is not formed, and thus the downstream synTF is not expressed. AD activation domain, *BS* binding site, DBD DNA binding domain, *P* synthetic promoter, *synTF* synthetic transcription factor, *T* yeast terminator.

For sustainable and reliable NP production, heterologous enzymes must be expressed after the biomass accumulation phase^66^. To date, exogenous chemical inducers (e.g., L-rhamnose, IPTG, and arabinose) have been used to control gene expression^67^. However, the application of chemical inducers is limited owing to hypersensitivity and toxic effects^68^. Therefore, the development of cost-effective inducers that enable modulation of protein expression levels in a fast-acting and robust manner would support stable biomanufacturing of pharmaceutical drugs^69^. In the context of the proposed platform, an optogenetic system is required to regulate the expression of synTFs. A customizable synthetic DNA-binding domain (DBD), such as a transcription activator-like effector^70^, to target the upstream promoter of synTF and a yeast activation domain (AD) to activate transcription are, respectively, linked with the light-using domain and its interacting partner domain (Fig. 5). The DBD binds the promoter of synTF regardless of the light condition. However, dimerization of light partner domains, allowing for activation of synTF, is light-induced (Fig. 5a). The synTF targets a synthetic promoter controlling the expression of a gene of interest. In the absence of light, the interaction of the light sensor and its partner is abolished, thus resulting in the disassociation of DBD and AD (Fig. 5b). Since blue light is required for C_1_-negative manufacturing, a system based on the red light-sensitive dimerization of the photoreceptor PhyB and its interacting partner, PIF3, may hold the promise^71^. Benzinger and Khammash have developed a stochastic ordinary differential equation-based gene expression model to predict the output of blue light-mediated gene expression^72^. Moreover, they have demonstrated how pulsatile blue light signaling allows graded expression of multiple genes at fixed expression ratios, regardless of differences in promoter dose-response features^72^. Using the same principle, pulsatile red light-induced temporal control can be established to optimize gene expression of NP biosynthetic pathways.

## System optimization

Evolution has been optimizing the expression level and the function of the enzymes to improve the structure of NPs for definite biological functions, such as cellular defence mechanisms and communication with other organisms^5^. To generate yeast cells with non-natural features, careful attention must be paid to balance the expression level of the required enzymes and to speed up their catalytic efficiency^73^. In addition, capturing cell resources for heterologous pathway gene expression may interfere with critical cellular processes and result in energetic inefficiency in the cell^74^. As a consequence, the enzyme activity and yield of the desired product are negatively influenced^74^. To overcome these challenges, approaches for rewiring cellular networks and synthetic evolution in the context of microbial production of NPs are needed.

### Rewiring cellular network

A considerable hindrance in microbial bioprocess scale-up for NP production is that the accumulation of NPs within intracellular membranes decreases cell growth (and consequently the yield of the desired NP)^75^. Another issue arises from the lower catalytic efficiency of plant NP enzymatic reactions in microbial cells compared to that in plants^73^. A strategy to overcome the aforementioned challenges involves targeting membranes of cellular organelles such as mitochondria, peroxisomes, and vacuoles for compartmentalization and the storage of synthesized NP. In addition, placing the heterologous enzymes inside the cellular compartment enables enzymes such as monoterpene synthases (which have lower catalytic efficiency than the enzymes involved in the cytosolic ergosterol pathway) to access their substrates^18^. Increasing the ER size to cope with P450 protein folding and accumulation has yielded a concentration of 31.01 mg/L for parthenolide, a precursor for the anti-glioblastoma drug ACT001^76^. In another study, to maximize the peroxisome membrane storage capacity of *S. cerevisiae*, the number and size of peroxisomes have been increased through the expression of peroxisome biogenesis-associated peroxins^77^. The peroxisomal compartmentalization of acetyl-CoA, coupled with increased expression of sesquiterpenoid synthase and redirected precursor supply from the mitochondria to the cytosol, has increased production of terpene (+)-valencene 549-fold (1.2 g/L)^74^. Dusseaux et al. have introduced the mevalonate pathway into the peroxisomes of *S. cerevisiae*^32^, which results in 125-fold greater plant terpene limonene production compared to that achieved in cytosolic production. Srinivasan and Smolke have reported an engineered yeast for increased production of hyoscyamine and scopolamine through chemical engineering of cytosol, mitochondrion, peroxisome, ER membrane, and vacuole^21^. The expression of four plant transporters, engineering cofactor regeneration mechanisms, and optimized growth conditions have resulted in an increase in de novo tropane hyoscyamine production of more than 100-fold (480 μg/L)^78^. Overall, depending on the complexity of the NPs^79,80^, different modifications should apply to the yeast strain to enhance precursor level, provide energy supply, and deliver an adequate level of NAD(P)H.

### Synthetic evolution

The balance of metabolism in yeast cells has been optimized by natural evolution, wherein natural selection for desired traits creates iterative rounds of genetic diversity-selection cycles over time^81^. To achieve the optimal level of NP production in yeast, besides rewiring the native global regulatory networks, the optimal expression level of heterologous enzymes must be evaluated. Therefore, many genetic diversities must be created and tested, in so-called synthetic evolution inspired by nature, which is a time-consuming task^82^. Here, I focus on combinatorial optimization methods to simultaneously assemble millions of variants, followed by high-throughput screening of optimal variants, to enable rapid synthetic evolution.

### Combinatorial optimization library

Strong promoters of yeast genes are repetitively implemented for metabolic engineering purposes in *S. cerevisiae*, and *P. pastoris*, which may create a metabolic burden on the cell (thus negatively affecting growth)^66^. The absent or very low transcription levels of the main biosynthetic genes for complex metabolites plays an essential role in the failure to detect compound production in native or heterologous hosts^15^. These issues are more severe and challenging for the long biosynthetic pathways of NPs, particularly alkaloids, which are highly toxic metabolites for the host cell^19^. Fast-growing collections of synTFs for *S. cerevisiae*^67^ and *P. pastoris*^28^ provide a sophisticated solution to this problem, because they enable orthogonal, tunable, and inducible expression of the target gene(s) at desired processing time. The synTFs are well suited to the platform discussed herein, especially for controlling heterologous gene expression with a light inducer^27,83^. Using combinatorial optimization strategies, a library of synTFs can be assembled upstream of the individual genes of a biosynthetic pathway to create synthetic transcriptional units^69^. Combinatorially assembled units in artificial operons are integrated into the multilocus of the yeast genome for stable metabolite production. For example, nine synTFs have been assembled with four genes (to convert farnesyl pyrophosphate (FPP) to aromatic β-ionone) in only seven combinatorial cloning reactions ^27^. The resulting 36 synthetic transcriptional unit modules have been used to generate a genomically engineered yeast library, in which various cells produce different levels of β-ionone.

In alkaloid biosynthetic pathways, master builder P450s play key roles in generating the structural variety underlying the functional diversity of produced NPs^84^. Modifying substrate recognition sites of a P450 responsible for the first putative oxygenation step of the forskolin pathway has been found to increase its catalytic activity up to 6-fold in plants^85^. Davies et al. have reported optimized expression ratio between P450s G8H and its redox partner increases the production of the insect-repellent nepetalactone^86^. It has been shown that combined synthetic biology and protein engineering allows to identify a suitable cytochrome P450 to decorate the terpene scaffolds leading to produce 28 different noncanonical terpenes^87^. Concerning several ambiguities about obtaining catalytically active heterologous enzymes (naturally occurring in plants), yeast must be engineered with multiple gene isoforms to identify the optimal enzyme variant. Including different isoforms of various enzymes in the context of combinatorial optimization strategies enables automated optimization at the levels of transcription and translation, thus opening new routes to enhance NP production in yeast cell factories. As a starting point, Zhang et al. have optimized the production of nepetalactol in *S. cerevisiae* using screening a combinatorial library of four isoforms for iridoid synthases and cyclases^7^.

### Generalized strategies for biosensor development

Detecting enzymes that can improve yield is a crucial challenge in the microbial production of chemicals^69^. The problem is more pronounced when therapeutic NPs, the end product of long-biosynthetic pathways, are participants in combinatorial optimization techniques^88^. Moreover, stable scale-up of manufacturing processes faces a bottleneck due to reliance on low-throughput analytical methods for assessing strain performance^89^. The identification of genetically encoded transcription factor (TF) biosensors may provide alternatives to natural selection through fast screening of top-performing cells. Thus genetically encoded biosensors may potentially become part of a general platform for sustainable production of NPs. d’Oelsnitz et al. have developed a generalized approach to identify TF biosensors to detect plant NPs that are based on the TetR-family of multidrug-resistant TFs from *Salmonella typhimurium*, RamR, and its derivatives^89^. RamR derivative biosensor libraries containing more than 10^5^ members have been filtered into several high-performing variants in less than one week, and used to detect several benzylisoquinoline alkaloids (BIAs)^89^. TF-derived biosensors were subsequently used to translate the BIAs production level into a fluorescence signal. In addition, the TetR-family regulator CamR from *Pseudomonas putida* has been established to detect the monoterpene NPs^90^. These data altogether highlight the generalized abilities of the approach to detect various alkaloid-based NPs. This strategy can also be used to screen the intermediate metabolites produced by various isoforms of, for example, P450s and their partners within a combinatorial library to identify the most efficient enzyme isoforms. Another general method to generate biosensors for user-defined molecules has been developed by Beltran et al.^91^. They have used the plant abscisic acid receptor PYR1, with a malleable ligand-binding pocket, to evolve 21 sensors for a range of small molecules, including structurally diverse natural and synthetic cannabinoids^91^. Using the systematic evolution of ligands by exponential enrichment (SELEX) strategy, we are potentially able to screen a library of synthetic riboswitches for one with high binding affinity to a NP of interest^69^. Jang et al. have reported RNA aptamers for naringenin using SELEX^92^. In this study, the high-affinity naringenin-responsive riboswitches have been identified through applying in vivo selection route to the library by supplementing naringenin at different concentrations during enrichments. With the established NP biosensors, yeast cells producing high levels of NP within the combinatorial library can be detected and sorted with a flow cytometer for further optimization at bioreactor scale.

## Output optimization

Bioprocess engineering aims to address the poor productivity of biomanufacturing systems arising from the manual handling of samples, dynamically changing growth environments, external sources of noise, and a lack of control over the conditions experienced by cells before and during growth^93^. Cybergenetics involves advanced bioprocess engineering approaches that combine engineering and biology, and support the control of engineered metabolic pathways through computer interfaces and feedback controlling biological processes in real time. In this context, Chi.Bio^94^ and eVOLVER^95^ are two optogenetic-ready, open-source bioreactors that have been developed and made commercially available. The possibility of continuous cell culturing in automated bioreactors enables optimizing growth conditions for the top NP producers of the combinatorial library. ReacSight, a complementary strategy to enhance bioreactor arrays for automated measurements, provides a flexible instrument control architecture^96^. The combined platform of ReacSight and Chi.Bio has been used to demonstrate the real-time optogenetic control of gene expression and to achieve dynamic control of the composition of a two-strain consortium^96^. Similar strategies can be applied to evaluate the target metabolite production in yeast using the light-inducible synTFs and to establish optimal microbial consortia between yeasts and bacteria for carbon-negative manufacturing. In addition, the NP production output of the system can be quantified by real-time measuring the biosensor-derived fluorescent output using flow cytometry linked to an automated bioreactor. Finally, the protocol extracted from the in situ optimization of NP-producing yeast cells in bulk culture would enable unprecedented strategies for bioreactor operation.

## Dive into deep-learning data

Previous sections have discussed a platform to simplify the complex tasks of maximizing the synthesis of different NPs in microbial cells. To develop rational engineering strategies for producing rare NPs in the microbial cell, understanding the sophisticated feedback regulations controlled by the intermediate compounds is essential^97^. The multilayer optimization platform discussed herein may potentially generate a large dataset for developing DL algorithms with direct applications in biomanufacturing. Next-generation sequencing (NGS) approaches have revolutionized the identification of DNA diversity^98^. NGS can unlock new ways to tackle the DNA diversity in not only top producers but also subpopulations of low- to non-producer cells within the (combinatorial) library. Nevertheless, it alone is not appropriate for identifying the combination of DNA sequence of synTFs and genes encoding enzymatic isoforms within a combinatorial library of yeast population. The high similarity of DNA sequences encoding various synTFs (identical activation domains, minimal promoters, and terminators are typically used to generate synTFs) makes it challenging to infer the relative combination of DNAs of synTF and enzymes during assembly analysis of a (sub) population^69^. The droplet-based single-cell RNA sequencing allows flag-tagging of individual cells within the population^99^. The detailed description of droplet-based single-cell RNA sequencing to evaluate a yeast NP-producing library is illustrated in Box 1. However, a drawback to single-cell RNA sequencing is the missing values or dropouts that often occur, mainly because of the low amount of starting material (and consequently failed amplification of the original RNAs)^100^. Nanopore sequencing produces reads as long as 10 kb, thus resolving the sequencing issue for the repetitive region of synTFs used to modulate the production of NPs. Analytic tools that consider the characteristics of long-read data are mandatory^101^, but the development of such novel tools might take time. Large-scale single-cell genomics and transcriptomic datasets can be potentially generated using RNA sequencing for both undesirable and desirable populations. Together with induction, production, and bioprocessing optimization data, these data can feed DL algorithms to fit mathematical models, including model predictive control algorithms^102,103^. Consequently, upon tight control of the experimental conditions and properly processing the generated data, the behavior of the biological system (e.g., dynamics of the gene expression) can be quantitatively described, and its functionality can be enhanced. Ultimately, the mechanisms that trigger loading problems are pinpointed and emergence of non-producer mutants is prevented in scaled-up fermentation.

How DL algorithms may specifically contribute to extracting maximum information from an extensive combinatorial library of NP-producing variants warrants an in-depth review. To call synthetic biologists and bioinformaticians to action in this regard, promising DL programs that are applicable to substantially improving NP production are suggested here. First, Enformer can learn to predict promoter-enhancer interactions directly from the DNA sequence^104^, thus allowing the design of more robust synTFs for bioengineering heterologous pathways. Second, an array of machine learning methods and amino acid property descriptors are applicable to protein redesign problems, essential tasks in NP biomanufacturing^105^. Third, the PRISM 4 platform can predict chemical structure^106^. Through combinatorial optimization of NP pathway engineering in yeast, new derivatives of NPs might be produced for new drug discovery. However, the isolation of novel NP molecules is hindered by the challenge of connecting sequence information to the chemical structures of the molecules. PRISM 4 enables the development of machine-learning methods to predict the likely biological activity of new encoded molecules. In addition to new DL algorithms, powerful computational hardware would be essential to improve the quality and reproducibility of small molecule studies. An important subject that must be addressed is the lack of accessibility of such data and their maintenance. More than 120 natural product databases and collections have been published since 2000^107^. However, many of them are not automated facilities and do not provide open access to researchers. Generating the dataset based on the FAIR data principle (Findable, Accessible, Interoperable, and Reusable)^108^, followed by uploading the dataset to third-party facilities such as CloudLabs (https://cloudlabs.us/) or biofoundries (https://agilebiofoundry.org/, http://syncti.org/synthetic-biology-foundry/), can accelerate the design-build-test-learn cycle of NP metabolic engineering in microbial cell factories^109^.

Box 1 Schematic of gDNA capture during RNA-seq of a combinatorial library Within the combinatorial libraries, each synthetic DNA cassette encoding synTF, and pathway genes in a single cell can conceivably be labeled with unique barcodes. **a**, “Barcode 1” is added to the terminator region of the synTF-encoding sequence, whereas a fragment containing artificially added “*CAP*_dT_” and “Barcode 2” is inserted upstream of the synTF binding site. The 10x Genomics gel beads consist of a pool of primer sequences that are used to separately index each cell’s transcriptome. The presence of *CAP*_dT_ in all primer sequences enables RT of the cellular mRNAs within a library. All primers on a single gel bead contain identical *CBC* sequences to identify all transcriptomes of a single cell. Each primer has a unique UMI to tag each individual *cDNA* fragment. **b**, The Chromium Controller from 10x Genomics enables generation of tens of thousands of labeled single-cell partitions in emulsion droplets within minutes. Each droplet contains an identifying barcoded gel bead. **c**, In each droplet, the gel beads and cells are dissolved. All fragments from the same cell share a standard 10x barcode. In the given example, “Barcode 1” and “Barcode 2” enable identifying the combination of *synTF* 1 and *CDS* 1; “Barcode 3” and “Barcode 4” enable identifying the combination of *synTF*2 and *CDS* 2. *UMI*s 1, 2, 3, and 4 enable molecular counting of the captured *mRNA* molecules *synTF* 1, *CDS* 1, *synTF* 2, and *CDS* 2, respectively. After reverse transcription, the oil is removed, and all tagged cDNA reads are pooled to be sequenced (alongside whole single-cell transcriptomics) with NGS. The turnkey bioinformatics tools map sequencing reads back by using the identifying barcodes. Abbreviations: *CAP*_dT_, capture poly dT; *CBC*, cell barcode capture; *cDNA*, coding DNA; *CDS*, coding DNA sequences; *gDNA*, genomic DNA; NGS, next-generation sequencing; RT, reverse transcription; *synTF*, synthetic transcription factor; *UMI,* unique molecular identifier.
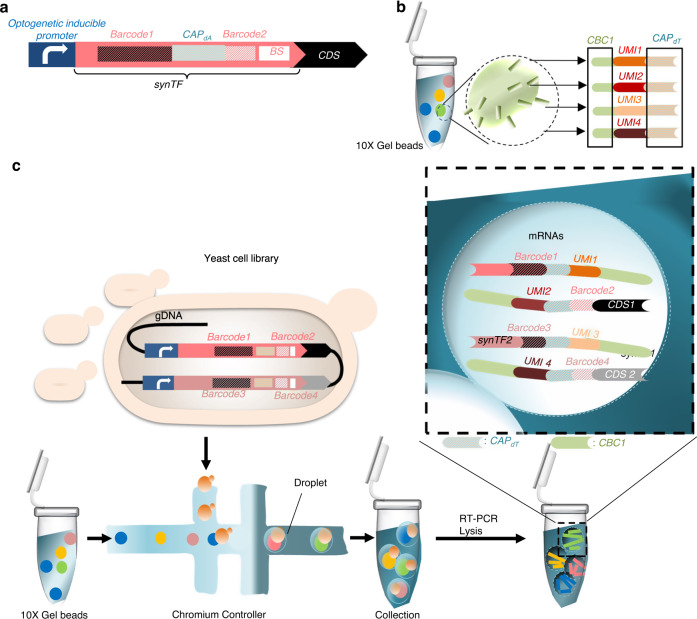

## Concluding remarks

We will face the consequences of the largest population explosion in human history in this century^110^, leading to demand for increasing cropland that competes with the need for land to produce plant-made pharmaceuticals. In a step toward sustainable manufacturing, a desirable strategy is to switch to low-carbon or carbon-neutral manufacturing. Some progresses have been made in converting C_1_ to value-added chemicals at the industrial scale by acetogens. For example, a recent study has suggested a promising approach for carbon-negative manufacturing of isopropanol chemicals at a large scale by harnessing autotrophic acetogens through metabolic engineering^111^. However, the conversion of C_1_ gases to complex NPs of plant, like MIAs, BIAs, by engineering acetogens has still been hindered mainly by the limited availability of genetic manipulation tools, compared to a variety of advanced tools available for yeast^36^. The slow growth and low productivity under autotrophic conditions are other barriers preventing the commercialization of acetogens on the industrial scale^112^.

To face possible difficulties to achieve sustainable NP production, a generalized microbial biomanufacturing platform using a wide range of state-of-the-art tools and techniques, as discussed herein, is necessary. To practically use the tools and methods in various laboratories, we must consider the requirement for standardization efforts^113^. Encouraging chemical engineering laboratories to apply similar principles in designing their toolboxes and methods, for example, according to the BioBrick design rules, and delivering the parts to the BioBrick repository may be a large part of the solution^114^. To rapidly iterate and improve the NP production protocol, the discussed platform (Fig. 2) was equipped with lab-scale bioreactors for in situ optimization of the output of NP-producing yeast in bulk culture. The generated optimized bioprocessing protocols significantly leverage developing large-scale production strategies. In scale-up bioreactors, the applicability of optogenetic induction systems and the possibility of the mixed microbial co-culture are two specific features for consideration. Open infrastructures such as UNLOCK bioreactor platform^115^ may offer promising avenues toward scale-up production of the NPs using CO_2_ and optogenetic inducer. The flexible arrangement of different types of bioreactors of UNLOCK platform allows for high-throughput cultivation of mixed cultures in MSBC and further optimization of the upstream and downstream bioprocessing protocols. Notably, safely storing the multilayer metadata produced according to the FAIR guiding principles^108^ is of particular relevance for simultaneously ingesting, exploring, and analyzing using DL approaches.

Before transitioning NP production technology to the commercial production level, an essential consideration is developing effective regulatory compliance, besides establishing the scale-up protocol. One of the highest commercial potentials is to focus on carbon-negative biomanufacturing strategies, as they may offer promising global warming solutions while concurrently providing further economic benefits from cheap C_1_ feedstocks by producing high-value NPs^116^. Recent progresses, like establishing anaerobic biofoundries, leads to the fast growth of this path^117^. Nevertheless, analytic studies on the quality, stability, and safety of C_1_-derived NPs, and the potential effect of by-products on the efficiency of processing during fermentation^118^ should be seriously considered. Furthermore, to develop cost-effective, large-scale biomanufacturing, deliberating on the process cost and technical and economic performances of the entire supply chain is necessary. For example, 30–50% of the manufacturing costs of supplies are typically allocated to downstream processing^118^. In addition, to successfully establish the discussed platform, communication issues between the disciplines must be addressed. Therefore, organizing annual interdisciplinary conferences focusing on sustainable NP production and workshops teaching various aspects of the involved disciplines are of critical importance. Providing more funding opportunities to support interdisciplinary projects can be an impetus for collaboration between different disciplines. For example, the willingness of biopharmaceutical companies to potentially commercialize C_1_-derived products opens new doors to defray some of the research costs to continue the investigation on improving the potential of the C_1_ technology. As a breakthrough step toward facilitating the funding of interdisciplinary fields, ‘Executive Order 14081’ on advancing biotechnology and biomanufacturing was recently approved in the USA, which aims to encourage federal investment in biotechnology and biomanufacturing. Hopefully, more countries will also take into consideration to further support research on developing the interdisciplinary field of sustainable biomanufacturing of small-molecule drugs.

Several tools and approaches to establish the discussed platform, e.g., synTFs, combinatorial optimization techniques, light inducers, bioprocessing optimization strategies, and single-cell sequencing technologies, can be implemented individually to optimize production of NP drugs in yeast. They can also be adapted to other bioengineering projects to facilitate the production of high nutritional value proteins, enzymes, and vitamins. Overall, the proposed biomanufacturing platform’s roadmap provides a unique niche for academia-industry collaboration.
